# Over-the-Counter Medicine Attitudes and Knowledge among University and College Students in Brunei Darussalam: Findings from the First National Survey

**DOI:** 10.3390/ijerph19052658

**Published:** 2022-02-24

**Authors:** Ishpa Shapiah Abdullah, Li Ling Chaw, David Koh, Zahid Hussain, Khang Wen Goh, Asma A’tiyah Abdul Hamid, Long Chiau Ming

**Affiliations:** 1 PAPRSB Institute of Health Sciences, Universiti Brunei Darussalam, Gadong BE1410, Brunei; ishpa.shapiah@gmail.com (I.S.A.); david.koh@ubd.edu.bn (D.K.); 2 Faculty of Health, University of Canberra, Canberra, ACT 2617, Australia; zahid.hussain@canberra.edu.au; 3 Faculty of Data Science and Information Technology, INTI International University, Nilai 71800, Malaysia; khangwen.goh@newinti.edu.my; 4 Department of Pharmaceutical Services, Ministry of Health, Bandar Seri Begawan BE3119, Brunei; asma.abdulhamid@moh.gov.bn

**Keywords:** over-the-counter (OTC), cross-sectional survey, safe use, knowledge, attitude, practice, behaviour, habit, Brunei

## Abstract

Over-the-counter (OTC) medicine is defined as safe and effective for the general public to use, without seeking therapy from a health professional. As primary social media and internet users, university and college students are more likely to be exposed to unverified sources of health information. This study aims to assess the knowledge, attitudes, and behaviour of students at institutions of higher learning in Brunei with regard to the safe use of OTC medicines. A cross-sectional study was performed using a self-administered online questionnaire, adapted from the literature with additional information from the United States Food and Drug Administration (FDA) on the educational resources in understanding OTC medicine for consumers. The questionnaire consisted of 4 sections: demographic information, knowledge of OTC medicines, attitudes, and practice. Descriptive and inferential statistics were used for data analysis. A total of 335 students returned a completed questionnaire. The students had a mean knowledge score of 7.1 out of 9, with more than half (53.4%) having good knowledge (knowledge score > 7), and only a small percentage (5.7%) with poor knowledge. Almost all of the students (99.7%) had a positive attitude toward OTC medicine use. Few of the students practiced improper habits in terms of OTC medicine use, such as not reading the instructions or taking more than the recommended dose. Awareness of proper OTC medicine use among students in institutions of higher learning is necessary to prevent the rise of inappropriate user practices.

## 1. Introduction

Over-the-counter (OTC) medicines are therapeutic products that pharmacies or retail outlets sell directly to consumers, unlike prescription medicines, which can only be dispensed based on a physician’s valid prescription [1]. OTC medications contribute to prevention and therapy for a broad spectrum of minor conditions, including but not limited to headaches, the common cold, musculoskeletal pain, allergies, and heartburn [2]. Usually, a regulatory agency chooses OTC medicines based on their safety and effectiveness profiles [3]. There are several important aspects of consumer practice of medication, including non-adherence, sharing of drugs, and self-medication. Self-medication involves a person purchasing medications to treat their symptoms without consulting a healthcare professional [4]. According to World Health Organization (WHO) drug information, self-medication is broadly accepted as an essential activity in healthcare with constant improvement in consumers’ general knowledge, education, and socio-economic status [5]. Self-medication is self-care in which a person can recognize their symptoms immediately and can usually treat these symptoms with medications that are easily accessible, such as OTC medicines [6].

Globally, inappropriate self-medication with OTC medicines has increased and is recognized as a public concern [7]. Misuse of various painkillers, vitamins, and sedatives have been reported among university students, and OTC drug misuse has become more popular among young adults [8,9]. Once these young people become older, they are responsible for making independent choices on obtaining and taking medication for their well-being [10]. Potentially inappropriate medication use from self-medication may occur due to a lack of knowledge [11]. This may include use for a wrong indication and administration of incorrect dosages [12].

Several studies have shown that university students lack knowledge of OTC medicine use and have poor attitudes toward proper medicine handling and safe use [13,14]. Poor knowledge and incorrect use of OTC medicine will lead to health risks such as adverse side effects, drug interactions and overdose. Based on information from the National Hospital Morbidity Database in Australia, young adults appear to be at high risk of paracetamol-related harm, with 4577 cases of paracetamol toxicity being recorded in 2017 [15]. Paracetamol was the most frequently used in overdoses, and a 108% increase in acute liver toxicity was reported between 2007 and 2017.

According to 2018 data presented by the Substance Abuse and Mental Health Services Administration, it is estimated that 6.5% of people aged 18 to 25 years old have misused OTC cough and cold medicines containing dextromethorphan (DXM) in their lifetime [16]. When a person consumes such medication in large doses, it may cause hallucinations or dissociative symptoms. The misuse of OTC medicines could be harmful to the users, affecting their health in the same way as illicit drugs.

Across Southeast Asia, self-medication practice for OTC medicine use is common. The various economic classes and healthcare systems in Vietnam, Singapore, and Thailand have reported high prevalence rates of self-medication with OTC medicines [17,18,19]. In Vietnam, self-medication of OTC medicines is a common practice among highland residents, with a prevalence rate of 83.3% in the last 12 months [17]. The overall prevalence of self-medication with OTC medicines among the working age population in Thailand is 88.2% [20]. In Malaysia, the prevalence of self-medication with OTC medicine among female students in Universiti Sains Malaysia (USM) is 80.9% [21]. Unfortunately, there is no information on self-medication with OTC medicines in Brunei. However, cultural values may impact the prevalence rates. With the increase in medication consumption, it is necessary to study medication knowledge and behaviours in young adults, especially students, to understand the information needed on medication use [22]. 

Brunei Darussalam is a small country with a population of 453,600, according to 2020 estimates [23]. The country offers a single-payer system of universal healthcare, with its health services and funding mainly financed by the central government [24]. Even though free medical treatment and medication are provided, the general public sometimes practices self-medication for minor ailments, primarily with leftover medicines at home or medicines bought directly from pharmacies or grocery stores [4]. Most of the time, Brunei residents purchase OTC medicines according to their knowledge and experience on the effectiveness of the medication.

To the best of our knowledge, there is no documented Bruneian study assessing the use of OTC medicines. A survey targeting the younger population, especially those undergoing university or college education, will be helpful, because they are the primary users of social media and so are most likely to be exposed to unverified sources of health information. Furthermore, such a survey is needed because it may benefit public health policymakers and local health authorities as baseline data for future health promotion and intervention. Therefore, this study aimed to examine the safe use of OTC medication by determining the knowledge, attitudes, and behaviour with regard to OTC medications among the students in Brunei’s institutions of higher learning.

## 2. Materials and Methods

### 2.1. Study Design

This is a cross-sectional online survey conducted from March to April 2021 involving all students enrolled in all public institutions of higher learning in Brunei Darussalam. There are six such institutions, namely: Universiti Brunei Darussalam (UBD), Universiti Teknologi Brunei (UTB), Universiti Islam Sultan Sharif Ali (UNISSA), Kolej Universiti Pergururan Ugama Seri Begawan (KUPU SB), Politeknik Brunei (PB), and Institute of Brunei Technical Education (IBTE). Full-time or part-time students enrolling in any of these institutions in Brunei Darussalam, 18 years old or above, and either Bruneian citizens or permanent residents, were eligible to participate. 

### 2.2. Study Instrument and Distribution 

The study questionnaire was adapted from a survey conducted by Tesfamariam et al. [7]. Additional information from the United States Food and Drug Administration (FDA) on the educational resources in understanding OTC medicine for consumers was also applied [25,26]. The online questionnaire was provided in English language, as it is the primary medium of instruction at Brunei’s institutions of higher learning. Face validity and pretesting were conducted to ensure that the questions were understandable for the target population and suitable for the local setting. Internal consistency of the questionnaire was also determined using Cronbach’s alpha.

The final questionnaire consisted of four sections with a total of 38 questions: seven questions on demographic data, nine questions on knowledge, eight questions on attitude, and 14 questions on practice (Supplementary File S1: Survey Questionnaire Used). The knowledge section consisted of basic general questions, such as on the definition of OTC medication and some medications that cannot be taken concomitantly, including food and drug interactions. For the knowledge section, correct responses were scored as one mark, while incorrect and unsure responses were scored as zero. The total knowledge scores ranged from zero to nine, which were then categorized into “Good” (≥seven correct answers), “Moderate” (four–six correct answers), and “Poor” (<four correct answers). The attitude section was measured using a five-point Likert scale with the following response options: Strongly disagree, Disagree, Neither agree nor disagree, Agree, and Strongly agree. These options were scored, respectively, as one, two, three, four, and five. The positive and negative attitude depends on the cumulative attitude score, with a score of 20 and above considered as a positive attitude.

The online survey was distributed to all students at all public institutions of higher learning through email invitations sent to the students’ official email addresses at the participating universities and colleges. The email list was collated by the assistant registrars, with the email invitation consisting of an online survey link and a participant information sheet. An email reminder was sent following a two-week interval to increase the response. Sampling was not necessary because all eligible participants were invited and were therefore given a chance to participate.

### 2.3. Data Analysis

Data collected were summarized using descriptive statistics for the percentages, frequencies, median, and mean. Chi-square and Fisher’s exact tests were used to analyse the association between the demographic data and knowledge. R version 4.0.4 software and Microsoft Excel version 2019 were used for data analysis [27,28]. A *p* value of <0.05 was considered statistically significant.

### 2.4. Ethical Approval

The Joint Research Ethics Committee of the PAPRSB Institute of Health Sciences, Universiti Brunei Darussalam, granted full approval for this study (UBD/PAPRSBIHSREC/2020/135). Additionally, the data collected remained anonymous, and confidentiality was strictly maintained. A written consent form was provided by asking respondents to tick an option at the beginning of the online survey. Participation was on a voluntary basis. 

## 3. Results

### 3.1. Face Validity and Pre-Testing 

The face validity of the online questionnaire was assessed by a pharmacist, an epidemiology/biostatistics lecturer, and a physician from the PAPRSB Institute of Health Sciences, Universiti Brunei Darussalam, for readability, length, and relevance. A pretest was also conducted among 38 students, after which the responses were used to finalize the questionnaire. The internal consistency of the final questionnaire was determined using Cronbach’s alpha coefficient. Cronbach’s alpha values for the knowledge, attitude, and practice sections were 0.74, 0.73, and 0.73, respectively.

### 3.2. Demographic Data

A total of 335 respondents returned the completed questionnaire. The median age of the respondents was 22.0 years (SD = 3.0, range between 18 and 44 years). The characteristics of the respondents are displayed in Table 1.

### 3.3. Knowledge of OTC Medication

More than half of the respondents (53.4%) had good knowledge of the use of OTC medicine, with a mean total knowledge score of 7.1 out of 9 (Table 2). Students’ level of knowledge was significantly different in terms of academic degree (*p* = 0.028) and course of study (*p* < 0.001, Table 3). Respondents’ level of knowledge differed by academic degree level (*p* = 0.01), but not in terms of nationality or year of study. There was a statistically significant difference of knowledge scores between students on health science-related and non-health science-related courses (*p* < 0.001).

A health science-related course is defined as students who are health science majors studying health and disease in the human body, such as medicine, dentistry, pharmacy, biomedical, and nursing. A non-health science-related course is defined as students who are not health science majors studying health and disease in the human body, such as engineering, IT, religion, finance, design, and art.

### 3.4. Attitudes on the Use of Over-the-Counter (OTC) Medicines

Almost all respondents indicated a positive attitude toward the use of OTC medicine (Table 4). Most agreed that using OTC medication for self-medication is safe when used correctly (80.9%), and that OTC medication is convenient to obtain and use (82.1%). About half (50.1%) agreed that OTC medication can be used during pregnancy and while breastfeeding, but with caution, unless there is a warning label to avoid the medication during pregnancy or breastfeeding. No significant differences were observed when comparing attitudes among those with different demographic characteristics.

### 3.5. Respondents’ Practices Regarding Over-the-Counter (OTC) Medication Use

**Figure 1:**
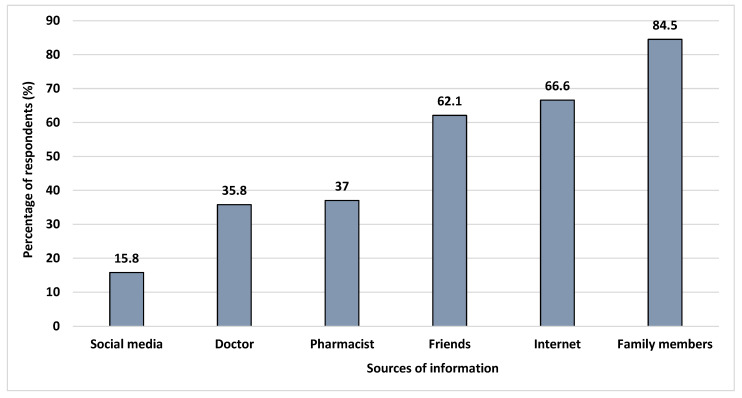
Sources of information considered by the respondents before purchasing an OTC medicine.

**Figure 2:**
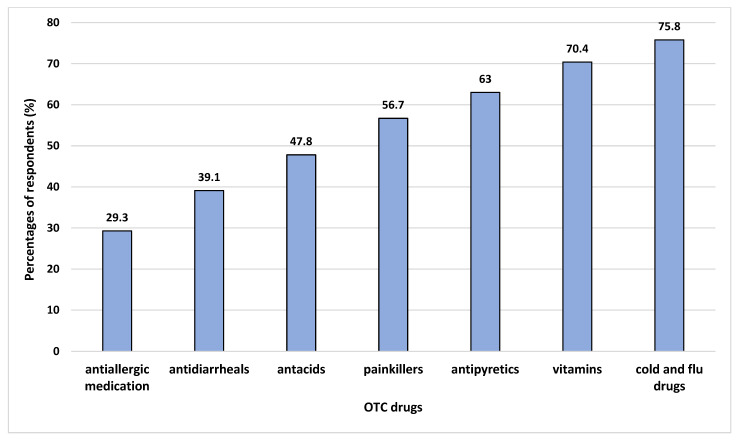
Respondents’ favoured OTC drugs.

The respondents depend on various sources of information before purchasing suitable OTC medicine, such as family members (84.5%), the Internet (66.6%), friends (62.1%), a pharmacist (37.0%), a doctor (35.8%), and social media (15.8%) (Figure 1). Their reasons for choosing OTC drugs are convenience (84.8%), ability to self-treat (79.7%), time saving (68.4%), low cost (n = 150, 44.8%), and safety (n = 143, 42.7%). The respondents’ favoured OTC drugs were cold and flu drugs (75.8%), vitamins (70.4%), antipyretics (63%), painkillers (56.7%), antacids (47.8%), antidiarrheals (39.1%), and antiallergic medication (29.3%) (Figure 2). 

A few (9.9%) of the respondents experienced adverse effects from OTC medicine (Table 5). In addition, 17.0% of the respondents did not know if they had ever experienced adverse events. Most respondents (83.3%) would never take more than the recommended dose, but 8.7% have consumed OTC medication over the recommended dose. Respondents reported that they had consumed over the recommended dose of paracetamol (Panadol) as the recommended dose had been ineffective. Reasons given by respondents for taking over the recommended dose included the body having adapted to taking more than the recommended dose, the medicine not being adequate, and being unaware of the dosage.

Most respondents always read the instructions on the medicine’s label before use (64.2%), check the expiry date (71.3%), and store the OTC drug in a cool, dry place or as stated on the label (60.0%). If the OTC medicine showed a change in shape, colour, or odour, 92.5% would immediately discard it.

## 4. Discussion

This study assessed the safe use of OTC medicine and determined the knowledge, attitudes, and practice around OTC medicine among students from public institutions of higher learning in Brunei Darussalam.

In this study, there was a significant difference between knowledge of OTC medicines depending on the academic degree (*p* = 0.028). There was also a significant difference between the knowledge of OTC medicine according to the course of study (*p* < 0.001). Health science-related students with a higher education level are more familiar with the clinical practice and medications, which improves their knowledge of OTC medicine use. Upper-year health science-related students have a better understanding of medications and self-medication since they are more exposed to the drugs than lower-year students. The finding of this study is similar to that from another study in Northwest Ethiopia, where fifth year students were found to be more likely to practice self-medication [29]. In Ethiopia, the mean knowledge score was 6.59, and 67.6% of the health science students had good knowledge of the safety and effectiveness of OTC medications [1]. More than 50% of the health science-related students had good knowledge, similar to Bruneian health science-related students (91.9%). Another study in Nepal showed that most students lack knowledge of self-medication [30]. Even though several studies showed that university students lack knowledge of the proper handling and safe use of OTC medicine, most students at English universities, and Universidade Federal do Rio Grande, Brazil were shown by respective studies to have good knowledge of the use of OTC medicines [13,14]. A good level of knowledge and proper use of OTC medicine may improve the health of students, as they will be more aware that some medications cannot be taken concomitantly, and also aware of food and drug interactions. It may also reduce the risk of adverse side effects and the economic burden on the government. 

We found no significant difference between the demographic data characteristics and the attitudes of respondents. Almost all students have a positive attitude toward the use of OTC medicine, with a mean attitude score of 27 out of 40. A high degree of OTC medicine consumption was apparent in 73.7% of study respondents. In Brunei, OTC medicines can be purchased and obtained directly, off the shelf, in stores, similar to in other Asian countries. This is common practice among university students in Vietnam, Malaysia, and India [17,31,32]. The majority buy cold and flu medicines, as the climates in Southeast Asia cause seasonal flu [33,34]. In Jordan and Belgium, analgesics and antipyretics were commonly used as self-medication among OTC products [35,36]. The majority of students asked their family before purchasing an OTC medicine, with the majority choosing cold and flu drugs. Moreover, in this study, those aged over 30 were more likely to purchase vitamins as self-medication, while those aged less than 30 were more likely to buy cold and flu drugs. In the United States, OTC analgesic use shows statistically significant increases among women aged 30 to 64 years old [37]. In South Korea, people aged 20 to 39 years old are less likely to use OTC medications for nutrition than those aged 19 years and younger [38].

The common reasons for choosing OTC for minor ailments were convenience, and to save time. These findings are similar to those from previous research in Germany and India, where the respondents reported that it saves time [39,40]. The most common source of information on OTC medicine was from family members (84.5%) rather than pharmacists (37.0%). This study reported that few students depend on information from social media (15.8%). This was an unexpectedly low level. In Papua New Guinea, students obtained information on OTC medicine from friends (37.2%) and family members (28.1%) [41]. In Nepal, the main sources of information referred to by medical students were pharmacists (60.3%), followed by textbooks (46.3%) and then advertisements (17.5%) [42]. Although the most common source of information is family members, pharmacists play a vital role in safeguarding the consumers from potentially inappropriate OTC medicine use [43]. 

Incorrect behaviours around self-medication with OTC medicine include taking more than the recommended dose, failing to check the expiry date, and rarely reading labels. In addition, continued consumption of OTC medicine with an unusual colour, odour, or shape changes is also inappropriate. About 5.7% of respondents reported that they rarely or never read the instructions on the medicine label. About 4.8% of the students responded that they rarely or never check the expiry date of the OTC medicine before using it. About 1 in 10 respondents (9.9%) reported having experienced adverse effects, while 17.7% did not know if they had ever experienced them.

Inappropriate practices of self-medication in developing countries have increased due to a lack of knowledge, inadequate medical information exposure, and confusing or unclear product labelling on different medicines [44,45]. Before using OTC medicine, reading the product label is the key to proper use, as it is taken without consulting a doctor. The findings of this study on checking the expiry dates are similar to those from a study in Eritrea where 73.9% of the respondents check the expiry dates [7]. However, 31% of respondents in Eritrea never read anything. This is different from the findings in this study, where just 0.9% of the Bruneian students never read the instructions on the medicine’s label. The results of this study on adverse effects are similar to those from the Philippines; 12% of the respondents aged over 18 with a college education had experienced adverse effects, while 11% did not know if they had ever experienced them [46]. Sometimes, the manufacturer makes changes to their OTC products, and that involves the label. This is concerning because not reading the label and checking the expiry date may lead to the build-up of those expired medications and many harmful effects [45]. 

Students are considered active participants in OTC medication as their knowledge influences drug use and the overall safety of drug use. Proper and appropriate self-medication practice can positively impact the individual and the healthcare system by saving lives from an acute ailment, reducing the waiting time in hospitals or clinics, reducing morbidity and decreasing healthcare costs.

### Limitations of the Study

This study has several limitations. Firstly, the questionnaire can be further improved to ensure it is more appropriate and relevant to the topic. One main reason is that the questionnaire was challenging to construct, and there were no comprehensive tools for testing the knowledge aspect available for this study. Secondly, the intentional misreporting of behaviours by respondents may confound the survey results or hide inappropriate behaviour. Hence, the responses to the questionnaire may not reflect the actual attitudes or behaviours of the respondents. Thirdly, as the practice segment of the questionnaire was embedded with recall questions on OTC medicine use, recall bias is likely to occur. Lastly, the male to female ratio was 25:75 in this study and 41:59 for overall higher institutions of learning in Brunei, indicating a potential oversampling of females in our study [46]. This could be due to non-responsiveness of the male student population, a factor that we could not control. Our study findings should be interpreted while taking the above points into consideration.

## 5. Conclusions

Students from institutions of higher learning in Brunei generally have a good level of knowledge and positive attitudes toward OTC drug use. There are still some gaps around inappropriate practices with OTC medicine, especially concerning reading of medication labels, checking of expiry dates and storage. However, only a minority of respondents have such inappropriate habits. Increased awareness of the proper use of OTC medicine among students in institutions of higher learning is necessary to prevent the rise of improper practices in OTC medicine use.

## Figures and Tables

**Table 1:** Demographic characteristics of study sample (*n* = 335).

Variable	*n* (%)
**Gender**	
Male	85 (25.4)
Female	250 (74.6)
**Nationality**	
Bruneian	328 (97.9)
Permanent Resident	7 (2.1)
**Higher institutions**	
Universiti Brunei Darussalam (UBD)	169 (50.4)
Universiti Teknologi Brunei (UTB)	28 (8.4)
Universiti Islam Sultan Sharif Ali (UNISSA)	36 (10.7)
Kolej Perguruan Ugama Seri Begawan (KUPU SB)	4 (1.2)
Politeknik Brunei (PB)	89 (26.6)
Institute of Brunei Technical Education (IBTE)	9 (2.7)
**Academic degree**	
Certificate (HNTec/NTec/skill certificate)	20 (6.0)
Diploma	80 (23.9)
Undergraduate	212 (63.3)
Master	16 (4.8)
Doctor of Philosophy (PhD)	7 (2.1)
**Course of study**	
Health science-related	111 (33.1)
Non-health science-related	224 (66.9)
**Year of study**	
Year 1	94 (28.1)
Year 2	105 (31.3)
Year 3	56 (16.7)
Year 4	74 (22.1)
Year 5	5 (1.5)
Year 6	1 (0.3)

IQR = interquartile range.

**Table 2:** Responses to knowledge of over-the-counter (OTC) medications (*n* = 335).

Statement	Correct	Incorrect	Unsure
	*n* (%)	*n* (%)	*n* (%)
OTC medicines are medicines you can buy without a prescription.	273 (81.5)	15 (4.5)	47 (14.0)
We are allowed to use OTC medicines to improve our health.	264 (78.8)	18 (5.4)	53 (15.8)
OTC medicines are used to treat, prevent or relieve major illnesses (brain and heart diseases).	217 (64.8)	33 (9.9)	85 (25.4)
OTC medicines are used to treat, prevent or relieve minor illnesses (fever and mild headache).	316 (94.3)	0 (0)	19 (5.7)
Interactions involving OTC medicines can sometimes produce unwanted results or make medicines less effective.	234 (69.9)	8 (2.4)	93 (27.8)
Some OTC medicines can also interact with foods and beverages and health conditions (high blood sugar and high blood pressure).	212 (63.3)	9 (2.4)	114 (34.0)
Pregnant and breast-feeding women should be extra cautious while using OTC drugs.	301 (89.9)	1 (0.3)	33 (9.9)
Painkiller is an example of an OTC medication.	286 (85.4)	12 (3.6)	37 (11.0)
Paracetamol (such as Panadol) is safe and effective when used correctly but taking too much can lead to liver damage.	278 (83.0)	2 (0.6)	55 (16.4)

**Table 3:** Association of demographic characteristics with level of knowledge (*n* = 335).

Characteristics	Knowledge Score			*p* Value
	Good ^1^	Moderate ^2^	Poor ^3^	
	*n* (%)	*n* (%)	*n* (%)	
**Gender**				0.127 ^a^
Male	53 (62.4)	24 (28.2)	8 (9.4)	
Female	179 (71.6)	60 (24.0)	11 (4.4)	
**Nationality**				
Bruneian	225 (68.6)	84 (25.6)	19 (5.8)	0.282 ^a^
Permanent Resident	7 (100)	0	0	
**Academic degree**				
Certificate	13 (65.0)	4 (5.0)	3 (15.0)	
Diploma	45 (56.3)	27 (33.8)	8 (10.0)	
Undergraduate	153 (72.2)	51 (24.1)	8 (3.8)	0.028 ^a^*
Master	15 (93.8)	4 (25.0)	0	
Doctor of Philosophy	6 (85.7)	1 (14.3)	0	
**Course of study**				
Health science-related	102 (91.9)	9 (9.9)	0	
Non-health science-related	130 (58.0)	75 (33.5)	19 (8.5)	<0.001 ^b^*

^a^ Fisher’s exact test ^b^ Chi-square test for independence * Statistically significant *p* value ^1^ Good = 7–9 marks; ^2^ Moderate = 4–6 marks; ^3^ Poor ≤ 4 marks.

**Table 4:** Attitudes of respondents to the use of OTC medicines.

Statement	*n* (%)
**Using OTC medicines as self-medication is safe when you use them correctly.**	
Strongly agree	121 (36.1)
Agree	150 (44.8)
Neither agree nor disagree	52 (15.5)
Disagree	11 (3.3)
Strongly disagree	1 (0.3)
**OTC medicines are convenient to obtain and use.**	
Strongly agree	160 (47.8)
Agree	115 (34.3)
Neither agree nor disagree	55 (16.4)
Disagree	5 (1.5)
Strongly disagree	0 (0)
**OTC medicines can be used in pregnancy and breastfeeding but with caution unless stated on the label to avoid**.	
Strongly agree	62 (18.5)
Agree	106 (31.6)
Neither agree nor disagree	97 (29.0)
Disagree	42 (12.5)
Strongly disagree	28 (8.4)
**I should take OTC medicines when I have minor illness**.	
Strongly agree	51 (15.2)
Agree	116 (34.6)
Neither agree nor disagree	124 (37.0)
Disagree	35 (10.4)
Strongly disagree	9 (2.7)
**OTC medicines are safe, but I would seek a pharmacist’s advice if I am not sure about my minor illness and which is suitable for it.**	
Strongly agree	206 (61.5)
Agree	74 (22.1)
Neither agree nor disagree	45 (13.4)
Disagree	7 (2.1)
Strongly disagree	3 (0.9)

**Table 5:** Respondents’ actions on the use of OTC medicine.

Statements	*n* (%)
**Have you ever practiced self-medication with OTC medicine(s)?**	
Yes	247 (73.7)
No	58 (17.3)
Do not know	30 (9.0)
**When do you usually consume OTC medicine(s)?**	
Symptoms are minor or manageable	226 (67.5)
Whenever I feel sick	194 (57.9)
Whenever I cannot visit doctor	212 (63.3)
**Have you experienced adverse effects from the OTC medicine(s)?**	
Yes	33 (9.9)
No	245 (73.1)
Do not know	57 (17.0)
**Have you ever taken more than the recommended dose for the OTC medicine(s)?**	
Yes	29 (8.7)
No	280 (83.6)
Do not know	26 (7.8)
**How often do you read the instructions on the medicine’s label before use?**	
Always	215 (64.2)
Often	60 (17.9)
Sometimes	41 (12.2)
Rarely	16 (4.8)
Never	3 (0.9)
**How often do you check the expiry date?**	
Always	239 (71.3)
Often	52 (15.5)
Sometimes	28 (8.4)
Rarely	12 (3.6)
Never	4 (1.2)
**How often do you store your OTC medicine(s) in a cool, dry place or as stated on the label?**	
Always	201 (60.0)
Often	90 (26.9)
Sometimes	32 (9.6)
Rarely	7 (2.1)
Never	5 (1.5)
**If the OTC medicine showed a change in shape, colour, or odour, I would immediately discard the medicine.**	
Yes	310 (92.5)
No	5 (1.5)
Do not know	20 (6.0)

## Data Availability

Not applicable.

## Supplementary Materials

The following supporting information can be downloaded at: https://www.mdpi.com/article/10.3390/ijerph19052658/s1, Supplementary File S1: Survey Questionnaire Used.
