# Supplementary material for: Over-the-Counter Medicine Attitudes and Knowledge among University and College Students in Brunei Darussalam: Findings from the First National Survey
Source: Int J Environ Res Public Health. 2022 Feb 24;19(5):2658. doi: 10.3390/ijerph19052658 (PMC8909889; doi:10.3390/ijerph19052658)
Supplement: Supplementary file 1 [file ijerph-19-02658-s001.zip › ijerph-1369852-supplementary.pdf]

Abdullah et al (2022). IJERPH

Over-the-counter medicine attitudes and knowledge among university and college students in Brunei Darussalam: findings from the first national survey

Questionnaire

Section 1: Demographic data

1. Gender:
   ☐ Male ☐ Female

2. Age (years):
   ____

3. Nationality:
   ☐ Bruneian ☐ Non-Bruneian

4. Ethnic group:
   ☐ Malay ☐ Chinese ☐ Indian ☐ Others: ________________

5. Which university or college are you from?
   ☐ Universiti Brunei Darussalam (UBD)
   ☐ Universiti Teknologi Brunei (UTB)
   ☐ Universiti Islam Sultan Sharif Ali (UNISSA)
   ☐ Kolej Universiti Perguruan Ugama Seri Begawan (KUPU SB)
   ☐ Politeknik Brunei
   ☐ Intitute of Brunei Technical Education (IBTE)
   ☐ Others: _______________

6. Major (Course of study):
   ____

7. Current year of study:
   ☐ Year 1
   ☐ Year 2
   ☐ Year 3
   ☐ Year 4
   ☐ Others: ________________________

Section 2: Knowledge

8. Medicines are always used on the prescription of a doctor.

   ☐ Yes
   ☐ No
   ☐ I do not know

9. All over-the-counter drugs (non-prescription or you can buy without prescription) are safe and effective. Examples of over-the-counter: flu and cough syrup, and Panadol.

   ☐ Yes
   ☐ No
   ☐ I do not know

10. Over-the-counter drugs are used for treating diseases like

   ☐ Hereditary diseases (passed on from one generation to another or in other words run in family)
   ☐ Minor illnesses and injuries
   ☐ I do not know

11. Over-the-counter drugs are approved for self-care.

☐ Yes
☐ No
☐ I do not know

12. Which of the following drugs fall under over-the-counter drugs? [will include the meaning and examples for each answer]

☐ Antipyretics
☐ Anti-cold
☐ Analgesics
☐ Anti-microbials

13. Over-the-counter drugs could be used after their expiry date.

☐ Yes
☐ No
☐ I do not know

14. Over-the-counter drugs can

☐ Sometimes cause side-effect(s)
☐ Mostly cause side-effect(s)
☐ Never cause side-effect(s)
☐ I do not know

15. While using over-the-counter drugs, caution should be taken mostly in

☐ Pregnancy
☐ Elderly
☐ Children
☐ Adolescent/middle adults

16. If suspected side-effect(s) are seen, then one should

☐ Immediately stop using the drug
☐ Take low dose until side effect(s) subside
☐ Continue taking the drug regardless the side effect(s)
☐ Report to a doctor or pharmacist
☐ Other: ________________________

17. All over-the-counter drugs when taken along with prescribed drug are safe.

☐ Yes
☐ No
☐ I do not know

Section 3: Attitude

18. Over-the counter drugs that are used for self-medication are safe.

☐ Strongly disagree
☐ Disagree
☐ Somewhat disagree
☐ Somewhat agree
☐ Agree
☐ Strongly agree

19. Over-the-counter drugs are cheaper and convenient.

☐ Strongly disagree
☐ Disagree
☐ Somewhat disagree
☐ Somewhat agree
☐ Agree
☐ Strongly agree

20. Paracetamol (Panadol) in overdose is a powerful poison.

☐ Strongly disagree
☐ Disagree
☐ Somewhat disagree
☐ Somewhat agree
☐ Agree
☐ Strongly agree

21. Over-the-counter drugs can modify or alter the action of another drug.

☐ Strongly disagree
☐ Disagree
☐ Somewhat disagree
☐ Somewhat agree
☐ Agree
☐ Strongly agree

22. All over-the-counter drugs can be used in case of pregnancy.

☐ Strongly disagree
☐ Disagree
☐ Somewhat disagree
☐ Somewhat agree
☐ Agree

☐ Strongly agree

23. Pain killers when taken on an empty stomach does not cause gastritis.

☐ Strongly disagree
☐ Disagree
☐ Somewhat disagree
☐ Somewhat agree
☐ Agree
☐ Strongly agree

24. Over-the-counter drugs are not affected by storage conditions, like temperature, moisture and direct sunlight.

☐ Strongly disagree
☐ Disagree
☐ Somewhat disagree
☐ Somewhat agree
☐ Agree
☐ Strongly agree

25. Liquid medicines could be used when opened after one month.

☐ Strongly disagree
☐ Disagree
☐ Somewhat disagree
☐ Somewhat agree
☐ Agree
☐ Strongly agree

26. Eye/ear drops could be used after one month of opening.

☐ Strongly disagree
☐ Disagree

☐ Somewhat disagree
☐ Somewhat agree
☐ Agree
☐ Strongly agree

27. It is better I do not take over-the-counter drugs when I am ill.

☐ Strongly disagree
☐ Disagree
☐ Somewhat disagree
☐ Somewhat agree
☐ Agree
☐ Strongly agree

28. Over-the-counter drugs are safe but would seek a physician advice before using it.

☐ Strongly disagree
☐ Disagree
☐ Somewhat disagree
☐ Somewhat agree
☐ Agree
☐ Strongly agree

Section 4: Practice

29. Have ever practiced self-medication with over-the-counter drugs

☐ Yes
☐ No

30. With whom did you consult before using over-the-counter drugs?

☐ Pharmacist
☐ Doctor
☐ Friends/relatives
☐ Leaflet
☐ Internet and mobile applications
☐ Other: ___________________

31. When did you consume over-the-counter drugs?

☐ When symptoms are minor/manageable
☐ Whenever I feel sick
☐ When I cannot visit doctor
☐ Other: ____________________

32. Common reason(s) for using over-the-counter drugs is:

☐ Time saving
☐ Low cost
☐ Safe and well tolerable
☐ Easy accessibility
☐ Other: _____________________

33. Which categories of medications are mostly preferred by you for self-medication? [will include the meaning and examples for each answer]

☐ Antipyretic
☐ Cough and cold preparation
☐ Analgesics
☐ Anti-inflammatory
☐ Anti-diarrheal
☐ Antacids
☐ Vitamin tablets
☐ Anti-allergic
☐ Other: ______________________

34. Have you experienced adverse effect from over-the-counter drugs?

☐ Yes
☐ No

35. What was the side-effect(s)?

____

36. What did you do after the side-effect(s)?

____

37. How often do you read the instructions on drug's label before use?

☐ Always
☐ Occasionally
☐ Rarely

☐ Never

38. How often do you check the expiry date?

☐ Always
☐ Occasionally
☐ Rarely
☐ Never

39. What do you do in case over-the-counter drug do not work well?

☐ Increase or double the dose
☐ Change to another powerful over-the-counter drug
☐ Straight away go to hospital or clinic
☐ Other: ________________________

40. If over-the-counter drugs showed change in shape, color, or odor:

☐ Immediately discard the drugs
☐ Continue using until it expires
☐ Continue using after it expires
☐ Other: ________________________
